# Placental Vascular Obstructive Lesions: Risk Factor for Developing Necrotizing Enterocolitis

**DOI:** 10.4061/2010/838917

**Published:** 2010-05-10

**Authors:** Laure Dix, Matthias Roth-Kleiner, Maria-Chiara Osterheld

**Affiliations:** ^1^Institute of Pathology, University of Lausanne, CH-1011 Lausanne, Switzerland; ^2^Division of Neonatology, Department of Pediatrics, University Hospital of Lausanne, CH-1011 Lausanne, Switzerland

## Abstract

Necrotizing enterocolitis (NEC) is a severe neonatal disease affecting particularly preterm infants. Its exact pathogenesis still remains unknown. In this study, we have compared the prevalence of vascular obstructive lesions in placentae of premature newborns which developed NEC and of a control group. We further compared separately the findings of placentae of infants of less than 30 weeks of gestation, the age group in which NEC occurs most frequently. We found signs of fetal vascular obstructive lesions in 65% of the placentae of preterm patients developing NEC, compared to only 17% of the placentae of preterm patients in the control group. In the age groups below 30 weeks of gestation, 58.5% of placentae of later NEC patients presented such lesions compared to 24.5% in the control group. The significant difference between NEC and control group suggests a strong association between fetal vascular obstructive lesions and NEC. Therefore, we propose that fetal vascular obstructive lesions might be considered as a risk factor for the development of NEC in premature infants.

## 1. Introduction

Necrotizing enterocolitis (NEC) is one of the most dreadful and unpredictable emergencies in premature infants [1, 2]. Its incidence is around 7% in very low-birth-weight infants (VLBW, birth weight <1500 g) [1, 3] and almost absent in full-term neonates [4]. The exact pathogenesis of NEC is still unknown. Many etiologic conditions have been described favorizing the development of NEC, such as gut immaturity, decreased gut motility, gastrointestinal bacterial colonization, and accelerated feeding [5–7]. Different authors suggested intestinal ischemia and hypoxia as important risk factors. The earlier proposed “diving reflex” in neonates suffering from severe hypoxic episodes with diversion of blood preferentially to heart, brain, and kidneys resulting in decreased perfusion of the intestinal tract could not explain all facets of the pathophysiology of NEC [8]. However, some pieces of evidence support the idea that hypoxic-ischemic events may play an important role in its etiology. (1) The ileocecal region which is often involved in NEC corresponds to an intestinal “watershed” area which might explain its susceptibility to hypoxic-ischemic events. (2) Reduced perfusion plays an important role in the pathophysiology of coagulation necrosis which represents one of the major histological findings of NEC [9]. (3) The rare condition of NEC in term infants is often associated with reduced intestinal perfusion secondary to congenital heart disease, patent ductus arteriosus, hypoxic-ischemic events, or polycythemia [10–12]. (4) Preterm infants showing a high-resistance flow pattern in the superior mesenteric artery on the first day of life were shown to be at higher risk to develop NEC later in their postnatal course [13]. These findings suggest that NEC might be associated with a disorder in regulation of splanchnic circulation in postnatal life. It has been shown in preterm infants developing NEC that their arginine level, a substrate for the synthesis of the import vasodilator nitric oxide, was reduced not only at the time of diagnosis [14] but already a week before the development of NEC [15]. Other situations which increase vascular resistance in the mesenteric arteries might be abnormalities of the development of the splanchnic circulation. Therefore, we hypothesized that a reduced mesenteric perfusion present already during fetal life might impede normal splanchnic vascular and gut development and create an increased vulnerability of the intestine. The most important causes of reduced fetal perfusion are maternofetal blood circulation disorders [16–19]. Chronic hypoxic-ischemic lesions due to placental insufficiency have been described to be most often the consequence of obstructive vascular lesions in the placenta, known as fetal vascular obstructive lesions [20].

We hypothesized that vascular obstructive lesions, representing a marker of maternofetal circulation disorders, might be overrepresented in placentae of neonates who will develop NEC in their postnatal course. In order to test this hypothesis, we have determined the prevalence of fetal vascular obstructive lesions in placentae of newborn infants who developed NEC and compared these findings with those of placentae of a control group.

## 2. Material and Methods

### 2.1. Patients and Study Groups

The discharge letters of all patients, hospitalized in the Division of Neonatology of the University Hospital of Lausanne, Switzerland, over an eleven-year period starting January 1994 were analyzed retrospectively with regard to the diagnosis of NEC. The Division of Neonatology in Lausanne is the only tertiary reference center for a region with about 12–14 000 births per year and accounts for about 500 annual admissions. Among all hospitalized patients, we identified 89 infants suffering from NEC (Bell stage II and more) [21, 22]. For these patients the archive of the Institute of Pathology of the University of Lausanne was screened. In 77 of the 89 cases (86.5%) the placenta had been sent to our institute and was available for reexamination. The placentae from the patients with NEC were considered the *study group*. As *control group* we could not use the total of placentae received during the same period because of technical problems due to a software change. Our institution received over the study period between 750 and 800 placentae per year. Considering that during eleven years only 89 patients with NEC were recorded, representing about 8 patients per year, the study group corresponded to about 1% of all the placentae received for analysis. According to these data, we have defined as control group the 769 placentae consecutively sent to our institution for histological examination during a one-year period (April 2006 to March 2007), an accepted methodology in the literature [23]. Indications for histological examination of the placenta in our area are maternal health problems like arterial hypertension, diabetes, hemorrhage or evidence of infection, intrauterine disorders like stillbirth or abnormal intrauterine growth as well as postnatal adaptation problems, prematurity, multiple births, and delivery by cesarean section. Placentae from completely normal pregnancies at term are usually not submitted for histological examination. Clinical information is provided by the demanding obstetrician or pediatrician on a clinical data form which includes information related to present and previous pregnancies, maternal diseases, clinical observations of the amniotic fluid, appearance of the placenta at delivery, information about the newborn as well as the indication for the examination. Due to the fact that NEC is mainly occurring in preterm infants (gestational age (GA) <37 weeks) and particularly in very preterm infants (GA <30 weeks), we did subsequent subgroup analysis taking into account only preterm and very premature infants labeled as *preterm control group (<37 weeks)* and *very preterm control group (<30 weeks).*

### 2.2. Placental Examination

All placentae were examined following a standard procedure as recently described [24]. In brief, all placentae were fixed for 48 hours in formalin in order to minimize artifacts due to different time lapses of fixation. Macroscopic findings such as abnormalities of structure, membranes (mother and fetal side), and umbilical cord were obtained and described. Placental weight was recorded after removing the umbilical cord and the amniotic membranes. Thereafter, the placentae were sliced every centimeter and lesions, if macroscopically evident, described and measured. Six to eight specimens were taken for microscopic examination from each placenta, including cord sections, and roll of membranes, cord insertion region, and at least three specimens from the placental parenchyma. The paraffin-embedded tissue blocks were then cut to a thickness of five microns, and routine histological staining was performed using hematoxylin and eosin. For study purposes, all placentae were reexamined independently by two different pathologists (LD and MCO).

### 2.3. Histological Definitions

**Figure 1:**
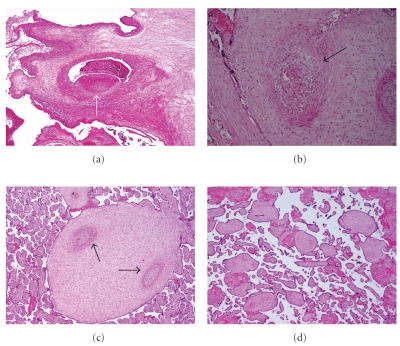
Characteristic histological findings of placental vascular obstructive lesions (all images colored by hematoxylin and eosin; magnification 40×). (a) Occlusive thrombus (arrow) in a dilated chorionic vein. (b) Hemorrhagic endovasculitis (HEV): extravasated blood cells (arrow) around a vessel in a stem villus. (c) Muscular hypertrophy and old occlusions (arrows) in stem vessels (obliterative endarteritis) (OE). (d) Avascular villi demonstrating hyaline villous stroma devoid of vessels.

Special interest was laid on the presence of fetal obstructive vascular lesions according to Baergen [25]. The presence of at least one of the different manifestations of obstructive lesions was defined as fetal vascular obstructive lesions. The lesions were documented for each specimen according to the following definition (Figure 1). *Thrombosis (IT and MT)*: presence of an isolated mural thrombus (IT) or multiple obstructive thrombi (MT) with or without complete obliteration of the vessel. Usually the veins are more frequently affected than the arteries. Prolonged occlusion by a thrombus mainly in the arterial circulation induces that the villous tree becomes avascular and atrophic. Therefore, we considered an avascular villi as direct evidence of thrombosis even without the possibility to localize a frank thrombus within the vessel. Avascular villi characterized by hyaline quality of the villous stroma were counted as IT for single ones or MT for multiple occurrence. *Obliterative endarteritis (OE)*: distal vessels presenting fibrotic thickening of the vascular walls (also called fibromuscular sclerosis), with or without obliteration of the lumen. *Hemorrhagic endovasculitis (HEV)*: microscopic criteria for the diagnosis of HEV include disruption or nonexudative necrosis of the vessel wall with hemorrhage, erythrocyte fragmentation, and intravascular nucleoplasmatic debris. HEV has been described in placentae from live newborns and stillborn fetuses and seems to be associated with an increase in perinatal complications [24].

### 2.4. Statistics

The study hypothesis was that the presence of placental vascular lesions might be significantly associated with the occurrence of NEC. Fischer's exact test was used to analyze correlations between the study and control groups. *P* < .05 was considered statistically significant.

### 2.5. Ethical Considerations

All patient data and pathological material were selected and handled according to the rules of the University Hospital of Lausanne, Switzerland.

## 3. Results

During the 11-year study period, 89 patients have been diagnosed with NEC (Bell stage II or higher). The study group consisted of 77 placentae which were available for reexamination. GA ranged between 23 6/7 and 36 2/7 weeks (mean: 28 4/7 weeks, median: 29 3/7 weeks). The average birth weight of the study group patients was 1520 g (range 950–2400 g) for those of GA between 30 and 37 weeks and 852 g (range 440–1840 g) for the NEC patients with GA < 30 weeks. The average birth weight of the investigated term neonates of the control group was 2669 g (range 1630–4250 g), 2187 g (range 1300–3140 g) for the preterm control infants between 30 and 37 weeks of gestation, and 693 g (range 306–1050 g) for the very preterm controls with GA < 30 weeks. 

The average weight of the placenta in the study group was 343 g (range 140–650 g) for the preterm pregnancies between 30 and 37 weeks and 206 g (range 90–221 g) for the very preterm pregnancies. The average placental weight of the 769 placentae in the control groups was similar: 421 g (range 242–935 g) for the term infants, 357 g (range 135–985 g) for the pregnancies between 30 and 37 weeks of GA, and 231 g (range 172–496 g) for the very preterm pregnancies (GA < 30 weeks). 

Macroscopically, the umbilical cord insertion was found in a comparable relative distribution in all the different groups: eccentric insertion in 50% of the study preterm group and 52.5% in the study very preterm group and in the controls: 57.5% in term placentae, 51.5% in preterm, and 49.2% in very preterm control placentae. Marginal insertion was described in 18.5% of the study preterm group, 25% of study very preterm group and in 17.4% in term controls, in 21.5% of preterm controls, and 19.4% of very preterm controls. Central insertion was found in a percentage between 12.5% and 23.9% in all groups and velamentous insertion was observed in a range of 4% to 13%. Interpositional insertion was only observed in 1% of the term control placentae. In the 77-later NEC patients only 5 cases (6.5%) of a false knot but no true knot were found. In the control group one true and 72 false knots (9.4%) were described. 

In one or more samples of 50 of the 77 placentae (64.9%) of the study group we found at least one of the four histological alterations of obstructive vascular lesions (Table 1). A total of 141 obstructive vascular lesions was observed in 130 of the analyzed 769 control placentae leading to a significantly lower prevalence of 16.9% in the control group. In the preterm subgroup, placentae of control newborns showed with 16.8% (47 of 279 cases) a significantly lower percentage of vascular obstructive lesions compared to the preterm study group (64.9%; 50/77). 

Overall, we observed 71 lesions in the 50 pathological placentae of the study group. The most frequent observation was multiple thrombi (MT) which were present in 30 (39.0%) of all placentae (Table 1). In the corresponding *preterm control group*, MT were rarer observations with a presence of only 5.4% (15/279 cases). All these results were statistically highly significantly different from those of the study group (*P* < .0001) (Table 1). The rest of the lesions like IT, OE, and HEV were also found more frequently in the study group compared to preterm control placentae.

The comparison of the subgroup of patients with the highest risk to develop NEC, the very preterm infants (<30 weeks), confirmed the above-mentioned differences. In the *very preterm study group* we diagnosed fetal vascular obstructive lesions in 24 of the 41 placentae corresponding to a prevalence of 58.5%. In the *very preterm control group* only 13 of 53 placentae showed one or more of these pathologies, representing a significantly lower prevalence of fetal vascular obstructive lesions with only 24.5% (*P* < .0001; Table 1). The pattern of lesions showed also a trend to more important pathologies in later-NEC patients, represented by a significantly higher number of MT lesions in the *very preterm study group* (34.1%) compared to the age-matched controls (7/53 (13.2%), *P* =  .023) (Table 1).

Chorioamnionitis and vasculitis may favor the formation of chorionic vessel thrombi. In order to omit this potentially confounding factor we analyzed the placentae with fetal vascular obstructive lesions regarding the presence of chorioamnionitis and/or vasculitis (Table 2). Although there was a high number of cases combining vascular obstructive lesions with chorioamnionitis and/or vasculitis, the above mentioned significant differences persisted with a higher prevalence of vascular obstructive lesions without chorioamnionitis and vasculitis in the placentae of future NEC patients compared to the controls (Table 2).

## 4. Discussion

NEC is a severe neonatal disease with a high degree of morbidity and an important mortality complicating the postnatal course of mainly premature newborn infants [1, 5, 26]. Although many etiologies proposed in the literature, the exact pathophysiological mechanism of NEC is still unclear. A multifactorial origin is very likely. Prematurity and low gestational age seem to be the most consistent risk factors [2, 3, 27]. Genetic predisposition and ethnic differences as well as altered or immature intestinal defense mechanisms and nutritional factors have been incriminated [5, 27]. Different studies suggested an implication of perfusion disturbances in the etiology of NEC like persistence and treatment modalities of ductus arteriosus or other pathological flow patterns in mesenteric arteries [2, 13]. The idea that placental insufficiency with a reduced perfusion pattern already in fetal life might have an impact on the occurrence of NEC was the topic of several studies and of a recent meta-analysis [28]. In fact, there seems to be an association between disturbed fetal perfusion patterns and the incidence of NEC [28, 29]. Although it is difficult to demonstrate a clear predictive value of pathologic fetal doppler measurements and the postnatal development of NEC, it might be reasonable to assume that a hypoxic-ischemic perfusion status of the fetus could contribute to some degree of vulnerability of the intestine leading to a predisposition to develop NEC later in postnatal life [28, 30]. 

Fetal vascular obstructive lesions are the result of stasis, hypercoagulability, and vascular damage within the fetal circulation of the placenta. They have been associated with fetal growth restriction and chronic fetal monitoring abnormalities. Placental lesions associated with fetal vascular obstruction have long been recognized and described using a variety of different names. Gruenwald was the first in 1961 to describe the clinical significance of avascular villi and their relationship to fetal thrombosis [31]. Others reported a high prevalence of disruptive fetal vascular lesions in term infants with adverse outcome and used the term of “hemorrhagic endovasculitis” to describe the spectrum of resulting placental changes [32–34]. Redline and Pappin highlighted the importance of even small foci of avascular villi in the absence of vascular thrombi and proposed the term of “fetal thrombotic vasculopathy” (FTV) [35]. This kind of placental lesions has been implicated in pathologies like cerebral palsy, fetal thromboembolic disease, intrauterine growth restriction, intrapartum monitoring abnormalities and discordant growth in twin pregnancies [19, 23, 36–41]. Severe fetal vascular lesions have been observed in over 50% of patients with hypoxic-ischemic encephalopathy or cerebral palsy at term [37]. Ogunyemi et al. have found among other pathologies a fourfold higher association of placental vascular abnormalities and NEC [42]. However, to our knowledge, any study so far has specifically evaluated the relationship between fetal vascular obstructive lesions and NEC. In this study we have compared the prevalence and the importance of obstructive vascular lesions in placentae of premature newborns which developed NEC and of a control group. We found a highly significant increase of fetal vascular obstructive lesions in placentae of newborns with NEC compared to the placentae of the control group. Previous studies have related placental pathology to preeclampsia, stillbirth, IUGR, and preterm labor suggesting that fetal vascular obstructive lesions might be responsible for prematurity but not directly for NEC [7, 22, 41–43]. Nevertheless, our results demonstrate a significant difference in prevalence and importance of fetal vascular obstructive lesions in placentae also among very preterm patients (<30 weeks of gestation). Those who developed NEC later in postnatal life had a significantly higher prevalence of fetal vascular obstructive lesions compared to age-matched control counterparts. These significant differences persisted also after correction for the presence of chorioamnionitis or vasculitis. Due to these findings of consistent association between fetal vascular obstructive lesions and NEC, in particular the presence of MT in the fetal placental vascular bed, we suggest that fetal vascular obstructive lesions have to be considered as an additional risk factor for NEC. As the result of the histopathological examination is available early in the postnatal course of a newborn infant, this information might alert the neonatologists in order to consider preventive or early therapeutic strategies.

In conclusion, we found an almost fourfold higher prevalence of fetal vascular obstructive lesions in placentae of future NEC patients compared to controls. We therefore strongly advocate a careful examination of placentae from premature babies in order to reveal the presence of fetal vascular lesions and, if present, to inform the neonatologists of the increased risk for the development of NEC.

## Figures and Tables

**Table 1:** FVOL: fetal vascular obstructive lesions defined as placentae with the presence of one or more of the following lesions: MT: multiple thrombi, IT: isolated thrombus, OE: obliterative endarteritis, HEV: hemorrhagic endovasculitis. *P*-value*: comparison between the three different control groups and the study group*  *P*-value**: comparison between the very preterm control group and the very preterm study group**.

	Total number of placentae	FVOL	Type of vascular obstructive lesions			
			MT	IT	OE	HEV
Study group*	77	50 (64.9%)	30 (39.0%)	15 (19.5%)	15 (19.5%)	11 (14.3%)

Very preterm study group (<30 weeks)^ ∗ ∗^	41	24 (58.5%)	14 (34.1%)	10 (24.4%)	5 (12.2%)	3 (7.3%)

Control group *P*-value*	769	130 (16.9%) .0001*	37 (4.8%) .0001*	59 (7.7%) .0021*	20 (2.6%) .0001*	25 (3.3%) .0002*

Preterm control group (<37 weeks) *P*-value*	279	47 (16.8%) .0001*	15 (5.4%) .0001*	27 (9.7%) .00268*	9 (3.2%) .0001*	7 (2.5%) .0002*

Very preterm control group (<30 weeks) *P*-value* *P*-value**	53	13 (24.5%) .0001* .0013**	7 (13.2%) .0015* .0237**	5 (9.4%) .1427* .086**	4 (7.5%) .0772* .049**	3 (5.7%) .1547* 1.000**

**Table 2:** Prevalence of chorioamnionitis (CA) and vasculitis in the different study populations. Comparisons are given between vascular obstructive lesions associated with chorioamnionitis, vasculitis, and without these inflammatory conditions. *P*-value*: comparison between the three different control groups and the corresponding study group. *P*-value**: comparison between the very preterm control group and the very preterm study group**.

	Number of placentae with CA	Number of placentae with vasculitis	Vascular obstructive lesions		
			With CA	With vasculitis	Without CA and vasculitis
Study group*	16 (20.8%)	14 (18.2%)	12 (15.6%)	9 (11.7%)	37 (48%)

Very preterm study group (<30 weeks)^ ∗ ∗^	12 (29.2%)	9 (21.9%)	7 (17%)	5 (12.2%)	19 (46.3%)

Control group (<37 weeks) *P*-value*	138 (17.9%)	101 (13.1%)	16 (2.1%) .0001*	5 (0.65%) .0001*	93 (12.25%) .0001*

Preterm control group (<37 weeks) *P*-value*	29 (10.4%)	34 (12.2%)	1 (0.36%) .0001*	1 (0.36%) .0001*	39 (14%) .0001*

Very preterm control group (<30 weeks) *P*-value* *P-*value**	32 (60%)	26 (49%)	5 (9.4%) .4373* .5420**	1 (1.9%) .0885* .0999**	7 (13.2%) .0031* .0150**
